# Fu Loose Tea Administration Ameliorates Obesity in High-Fat Diet-Fed C57BL/6J Mice: A Comparison with Fu Brick Tea and Orlistat

**DOI:** 10.3390/foods13020206

**Published:** 2024-01-09

**Authors:** Yan Liang, Fanhua Wu, Daying Wu, Xiaofang Zhu, Xin Gao, Xin Hu, Fangrui Xu, Tianchen Ma, Haoan Zhao, Wei Cao

**Affiliations:** 1 College of Food Science and Technology, Northwest University, Xi’an 710069, China; lianyan5517@126.com (Y.L.); wufanhua_1@163.com (F.W.); 18802945129@163.com (F.X.); 20200084@nwu.edu.cn (T.M.); zhaohaoan@nwu.edu.cn (H.Z.); 2 Key Laboratory of Fu Tea Processing and Utilization, Ministry of Agriculture and Rural Affairs, Xianyang 712044, China; zhuxf819@163.com (X.Z.); huxin@hotmail.com (X.H.); 3 Shandong Academy of Agricultural Sciences/National Engineering Research Center of Wheat and Maize/National Key Laboratory of Wheat Breeding, Ministry of Science and Technology/Key Laboratory of Wheat Biology and Genetic Improvement in North Yellow & Huai River Valley, Ministry of Agriculture/Shandong Provincial Technology Innovation Center for Wheat, Jinan 250100, China; wudaying2020@163.com (D.W.); bestgaoxin@126.com (X.G.); 4 Xianyang Jingwei Fu Tea Co., Ltd., Xianyang 712044, China

**Keywords:** Fu loose tea, Fu brick tea, obesity, lipid metabolism, inflammation, gut microbiota

## Abstract

Fu tea is receiving increasing attention for its specific aroma, flavor, and dramatic functional benefits. Herein, we explored the effects and underlying mechanisms of Fu loose tea (FLT), Fu brick tea (FBT), and diet pills (orlistat) on a high-fat diet (HFD)-induced obesity. The results indicated that FLT and FBT administration effectively inhibited weight gain, glucose metabolic dysregulation, fat accumulation in organs, hepatic and kidney injury, and oxidative stress induced by HFD. Additionally, FLT and FBT treatments improved the lipid profiles and reduced the production of proinflammatory cytokines by regulating the expression levels of lipid metabolism- and inflammation-related genes. Furthermore, FLT and FBT ameliorated the gut microbiota dysbiosis in HFD-mice in a dose-dependent relationship by increasing the abundance of family Verrucomicrobiaceae and genus *Akkermansia* and *Turicibacter* and simultaneously reducing the abundance of family Erysipelotrichaceae and genus *Bifidobacterium*; in contrast, orlistat did not exert a regulatory effect on gut microbiota similar to FLT and FBT to improve HFD-induced obesity. KEGG analysis of gut microbiota annotation revealed that “metabolism” was the most enriched category. This study further provides a theoretical basis for FLT and FBT to be potential supplements to alleviate diet-induced obesity.

## 1. Introduction

It has been widely evident that diet plays a critical role in developing several metabolic diseases. Owing to the changes in lifestyle under the current economic conditions, progressively escalating rates of overweight and obesity as a consequence of the high-fat diet (HFD) have become a severe public health issue worldwide, resulting in the emergence of a new term, “globesity” [1]. Around the world, the high body-mass index affects about 603.7 million adults, and it is shocking and alarming that over 60% of deaths can be attributed to complications of obesity [2]. Long-term HFD-induced obesity is characterized by fat accumulation in the adipocytes and overweight gain, which may elevate the risk of exposure to multiple metabolic diseases in individuals [3,4,5,6,7]. Even though diet pills can effectively lose weight, they lead to some severe drawbacks, such as short-term benefits, side effects, reduced immunity, and easy rebound [8,9]. Thus, it is necessary to prevent and alleviate obesity through safer and more effective interventions. In recent years, as awareness of healthy eating has grown, more consumers have focused on food’s nutritional and functional benefits [2]. Dietary interventions to alleviate obesity and various metabolic diseases without restricting energy intake have attracted increasing attention from researchers.

Tea (*Camellia sinensis*) has gained widespread popularity as a beverage for its distinctive flavor and health benefits [10]. Chinese commercial tea is traditionally divided into several categories based on the degree of fermentation: non-fermented, lightly fermented, semi-fermented, fully fermented, and microbially fermented [11]. As a trendy primary type of post-fermented tea, Fu tea (FT), including Fu brick tea (also named Fuzhuan brick tea, FBT) and Fu loose tea (FLT), is known for the existence of a dominant fungus named *Eurotium cristatum* (also called “golden flower fungus”) that is yellow-colored and gives the specific aroma and flavor to FT [12]. It dates back to the 13th century and was the most consumed among commercial tea varieties along the ancient Silk Road. FT is now widely produced in Shaanxi and Hunan provinces in China [13]. Because of its predominance in promoting digestion and cleansing the palate, FT has been consumed as a common beverage in the daily lives of nomadic groups in the border regions of southwestern and northwestern China who rely on meat and milk as their primary diet.

However, due to the time-consuming processing and inconvenient consumption of FBT in the form of large bricks during drinking, FLT, produced from tea in a loose state with non-pressing and short-duration fungal *E. cristatum* fermentation processes [14], can take advantage of an opportunity and meet the requirement for a convenience-oriented lifestyle. Increasing evidence has indicated that the health benefits of FBT may be attributed to the bioactive compounds, including polyphenols, alkaloids, and terpenoids [15], especially polyphenols, which are often reported for their health-promoting effects [16]. Different from the seaweed polyphenols consisting mainly of tannins (e.g., brown seaweeds characterized by phlorotannins) [17], tea polyphenols, as plant secondary metabolites, primarily include, but are not limited to, catechins, anthocyanins, phenolic acids, and flavanones, particularly catechins and their derivative compounds [18]. Previous studies have demonstrated that polyphenols and other bioactive compounds from FBT could alleviate multiple metabolic diseases, such as obesity [19,20], hyperlipidemia [21], and type 2 diabetes [22]. Nevertheless, no study discussed the effects of FLT on obesity induced by HFD and compared the differences in alleviating metabolic disorders between FBT and FLT.

With the promotion of economic globalization and the Belt and Road initiative, Shaanxi, as the starting point of the Silk Road, occupies a crucial geographical advantage. Thus, in recent years, Fu tea from Shaanxi, with its characteristic aroma and flavor, has been favored and consumed worldwide.

This study aimed to evaluate the effects of FLT and FBT administration (collected from Shaanxi, China) at different doses on obese mice induced by HFD. Hence, the anti-obesity characteristics of FLT and FBT were measured by examining the changes in lipid accumulation, metabolic disorders, oxidative stress, liver and kidney damage, inflammatory response, and the gut microbiota structure, which were also compared with the anti-obesity characteristics of diet pills (orlistat). This study not only demonstrated that FLT, as well as FBT, is an excellent supplement for alleviating HFD-induced obesity but also provided a new and comprehensive perspective for the prevention and alleviation of obesity caused by long-term HFD.

## 2. Materials and Methods

### 2.1. Sample Collection and Preparation

FLT and FBT were all acquired from Xianyang Jingwei Fu Tea Co., Ltd. (Xianyang, China). According to the previous study [23], the extracts were prepared by crushing, extraction, filtration, and vacuum concentration performed on FLT and FBT. After crushing, FLT and FBT were extracted twice using water (6.7%, *w*/*v*) at 100 °C for 10 min, then filtered with 200-mesh filters (75 μm). The filtrates were concentrated at 50 °C with a vacuum pressure of 0.09 MPa to obtain FLT and FBT extracts. The FLT and FBT extracts contain 3.08 ± 0.01% and 4.12 ± 0.02% free amino acids (FAAs), 15.23 ± 0.83% and 16.67 ± 1.01% soluble sugars (SSs), 3.62 ± 0.03% and 3.90 ± 0.03% caffeine (CAF), 10.70 ± 0.09% and 9.51 ± 0.07% tea polyphenols (TPs), and 1.53 ±0.01% and 1.47 ±0.02% total flavonoids (TFs), respectively. The FFAs, CAF, and TPs were determined according to the spectrophotometry method described in the National Standards of the People’s Republic of China GB/T 8314-2013 [24], GB/T 8312-2013 [25], and GB/T 8313-2018 [26], respectively. The SSs and TFs were measured using colorimetric methods based on anthrone [27] and aluminum chloride [28], according to the reported methods. The TPs and TFs were quantified based on the equivalent content of gallic acid and rutin, respectively. The extracts were stored at −20 °C before further analysis and utilization.

### 2.2. Animals and Experimental Design

**Figure 1:**
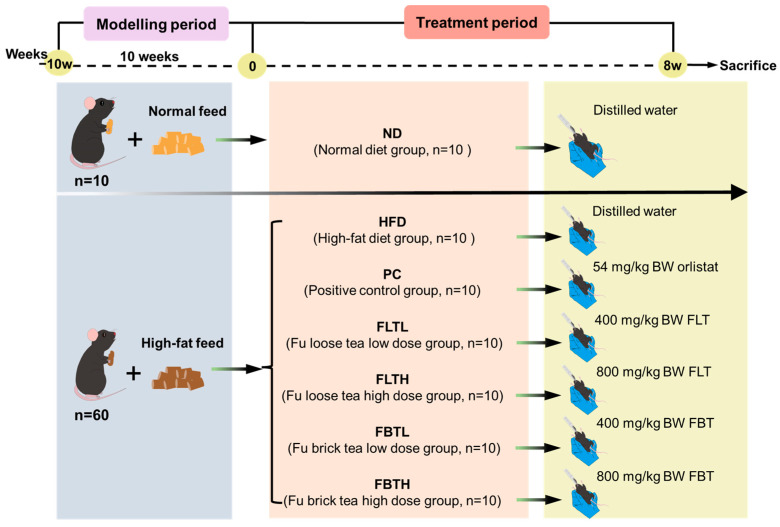
Experimental design.

The animal experiment was approved by the Animal Ethics Committee of the Laboratory Animal Center of Northwest University (NWU-AWC-20220503M). Seventy male pathogen-free C57BL/6J mice (5-week-old and body weight (BW) 21.17 ± 0.34 g) from the Beijing HFK Biotechnology Co., Ltd. (Beijing, China, SCXK<Jing>2019-0008) were raised in a room controlled with 22–25 °C, 50–60% humidity, and a 12 h light–dark cycle. Following one week of acclimation to the laboratory environment, the animals were randomly assigned to two groups: the standard diet group (ND, n = 10) received a normal diet comprising 10% calories from fat with an energy coefficient of 3.41 kcal/g, and the high-fat diet group (HFD, n = 60) received a diet containing 45% calories from fat with an energy coefficient of 4.42 kcal/g. After ten weeks, as shown in Figure 1, mice fed with an HFD were further randomly grouped into six groups (n = 10, two cages for each group) and were kept with an HFD but administrated with different supplements daily for eight weeks by oral gavage: the HFD group was supplemented with distilled water, the positive control (PC) group was administrated with 54 mg/kg BW orlistat, the FLT groups were supplemented with FLT at a dose of 400 mg/kg BW (FLTL) and 800 mg/kg BW (FLTH), respectively, and the FBT groups were administered with FBT at a dose of 400 mg/kg BW (FBTL) and 800 mg/kg BW (FBTH), respectively, while the ND group was still administered with a normal pelleted diet and was also supplemented with distilled water for eight weeks by oral gavage. The solution volume (fixed with water) of oral gavage for each mouse was 0.3 mL, and all mice were granted free access to fresh water and feeds.

The BW and food intake of mice in each group were monitored and recorded weekly and daily, respectively. After seven weeks of administering different supplements, the oral glucose tolerance test (OGTT) was conducted. Before OGTT, all mice experienced a six-hour fasting period, followed by oral administration of 2.5 g/kg D-glucose. The blood glucose levels were determined by tail bleeding at times 0, 30, 60, 90, and 120 min after D-glucose supplementation using a glucose meter (Sinocare Biosensing Co., Changsha, China). The area under the curve (AUC) was utilized as an evaluation parameter for the OGTT result.

After an eight-week experiment and being fasted for 12 h, mice were anesthetized to collect the blood from the orbital venous plexus. The liver, kidney, and epididymal white adipose tissue (eWAT) were collected and weighed as soon as the mice were sacrificed. The serum was obtained by centrifuging the blood. The sections of fresh liver tissues, eWAT, and kidney tissues for histological analysis were fixed in 4% formalin for 48 h at room temperature. The remaining samples and colon contents were stored at −80 °C for subsequent analysis.

### 2.3. Biochemical Parameters Analysis

The homogenizing liver tissues were centrifuged (4 °C, 822× *g*, 15 min), and the supernatant was taken for biochemical parameter analysis. Total cholesterol (TC), total triglyceride (TG), low-density lipoprotein cholesterol (LDL-C), high-density lipoprotein cholesterol (HDL-C), aspartate aminotransferase (AST), alanine aminotransferase (ALT), uric acid (UA), creatinine (CRE), blood urea nitrogen (BUN), glutathione (GSH), superoxide dismutase (SOD), malondialdehyde (MDA), and nitric oxide (NO) were measured by commercially available kits (Jiancheng Bioengineering Institute, Nanjing, China) following the provided instructions. In addition, tumor necrosis factor-alpha (TNF-α), interleukin-1-beta (IL-1β), and interleukin-6 (IL-6) were measured using mouse kits (Servicebio Technology Co., Ltd., Wuhan, China).

### 2.4. Histopathological Analysis

The formalin-fixed liver, eWAT, and kidney tissue samples were embedded in paraffin, sectioned at 3–5 μm, and stained with hematoxylin and eosin (H&E) for histopathological analysis. According to the previous study [29], the expression of hepatic nuclear factor-kappa B (NF-κB) p65 was analyzed by immunohistochemical analysis using the liver paraffin slices. The NF-κB p65 monoclonal antibody (1:200, Servicebio Technology Co., Ltd., Wuhan, China) and the horseradish peroxidase (HRP)-labeled goat anti-mouse IgG (1:200, Servicebio Technology Co., Ltd., Wuhan, China) were applied as the primary and secondary antibodies, respectively. Diaminobenzidine (DAB) was used as a chromogenic agent. The images of prepared-stained sections were observed by an optical microscope (TE2000-U, Nikon, Tokyo, Japan).

### 2.5. RNA Extraction and Real-Time Quantitative PCR (RT-qPCR)

To evaluate the expression of AMP-activated protein kinase (AMPK), sterol regulatory element binding protein-1c (SREBP-1c), c-Jun-N-terminal kinase (JNK), peroxisome proliferator-activated receptor-alpha (PPARα), acetyl-CoA carboxylase (ACC), phosphatidylinositol-3-hydroxy kinase (PI3K), fatty acid synthase (FAS), protein kinase B (AKT), and IkappaB-alpha (IκB-α) in liver tissue samples, total RNA was extracted according to TRIzol^TM^ kit (Invitrogen, Thermo Fisher Scientific Inc., Carlsbad, CA, USA) instructions. The RNA quality was assessed by NanoDrop 2000 (Thermo Fisher Scientific, Wilmington, DE, USA). The cDNA synthesis was carried out using the PrimeScript^TM^ RT Master Mix kit (Takara Bio. Inc., Takara, Japan). The gene expression levels were quantified via RT-qPCR, normalized to housekeeping gene glyceraldehyde 3-phosphate dehydrogenase (GAPDH), and calculated by the 2^−ΔΔCT^ method. The primer sequences are displayed in Table S1.

### 2.6. Gut Microbiota Analysis

Microbial DNA in colon contents collected from seven groups (n = 5) was extracted using a Soil DNA Kit (Omega Bio-Tek, Norcross, GA, USA). 16S rRNA sequencing was conducted based on the previous study [30] using the Illumina NovaSeq platform (Illumina, San Diego, CA, USA) to obtain 2 × 250 bp paired-end reads. After screening the raw reads and trimming the barcode, the high-quality reads were denoised by the QIIME2 data2 plugin [31]. The operational taxonomic units (OTUs) were with a 97% similarity cutoff. The selected sequences were aligned with the Greengenes database for species annotation. The number of taxonomic units presented at each level from phylum to species was counted for each group of the samples according to the species annotation results.

Alpha diversity analysis and beta diversity analysis (nonmetric multidimensional scaling analysis (NMDS)) were conducted to assess the variations in gut microbiota among different treatment groups. By aligning with the known reference genome data, the PICRUSt2 software (Version 2.4.0) was used to predict the functional abundance of the samples. Besides, the relative abundance data prediction of the metabolic pathways was carried out in each sample based on the KEGG Pathway Database (http://www.genome.jp/kegg/pathway.html accessed on 25 September 2023).

### 2.7. Statistical Analysis

All experiments were biologically conducted in triplicate. The data in this study were presented as the mean ± standard deviation (SD). Statistical analysis was performed using SPSS Statistics 17.0 software (SPSS Inc., Chicago, IL, USA) with one-way analysis of variance (ANOVA) and Duncan’s multi-range test for measuring the significance of differences between groups. The independent sample t-test was performed to analyze the significance between the two groups using GraphPad Prism 8.0.2 (GraphPad Software Inc., La Jolla, CA, USA). A *p*-value less than 0.05 indicated a significant difference.

## 3. Results

### 3.1. Effect of FLT and FBT on the Basic Parameters

**Figure 2:**
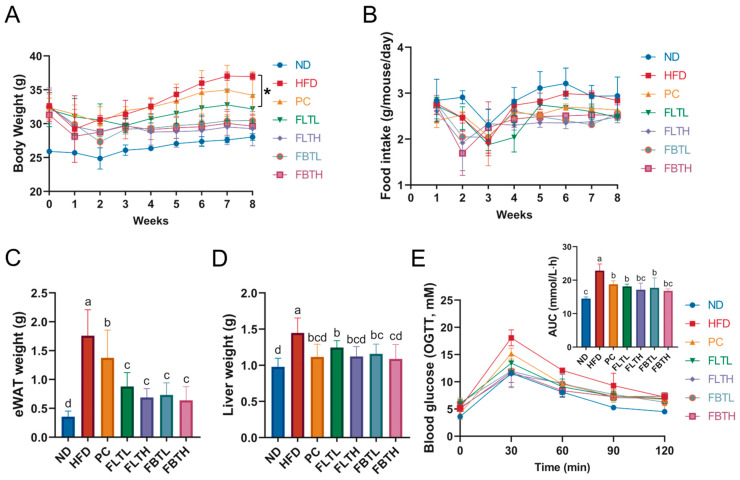
Effect of FLT and FBT on basic parameters in HFD-induced obese mice. (**A**) Body weight; (**B**) food intake, (**C**) eWAT weight, (**D**) liver weight, and (**E**) blood glucose level and its AUC during OGTT. Data are presented as means ± SD (n = 10 per group). * *p* < 0.05. Significance (*p* < 0.05) among groups is expressed by different letters.

Figure 1 displays the animal experimental scheme. After feeding with a standard diet or HFD for ten weeks, mice were administered different supplements daily for eight weeks by oral gavage. There was no death of C57BL/6 mice during the experiment, as well as good living and mental status. The changes in BW and food intake of the mice are shown in Figure 2. Compared with the ND group, mice with HFD intake consumed less food but gained more weight. No statistical significance was found in the average initial BW in HFD-fed mice before the different supplements were provided. During the first two weeks of oral administration, the BW of all groups decreased, which could be attributed to the stress response. In the subsequent treatments, the HFD group exhibited a sustained and rapid increase in BW, while mice fed HFDs in the PC, FLTL, FLTH, FBTL, and FBTH groups presented a decrease in BW gain. In particular, the FLTH and FBTH groups showed no significant difference in final BW compared with the ND group. The eWAT and liver weights of the PC, FLTL, FLTH, FBTL, and FBTH groups were lower than those of the HFD group (Figure 2C,D). In addition, OGTT was used to explore the effect of FLT and FBT on glucose tolerance in HFD-fed mice (Figure 2E). Compared with the rapid increase and slow decline of blood glucose in the HFD groups, FLT or FBT supplementation regulated and decreased blood glucose (significant reduction in AUCs). These results indicate that FLT and FBT could effectively alleviate weight gain, hepatic steatosis, adipocyte hypertrophy, and impaired glucose tolerance caused by long-term HFD.

### 3.2. Lipid Metabolism Analysis after FLT or FBT Administration

**Figure 3:**
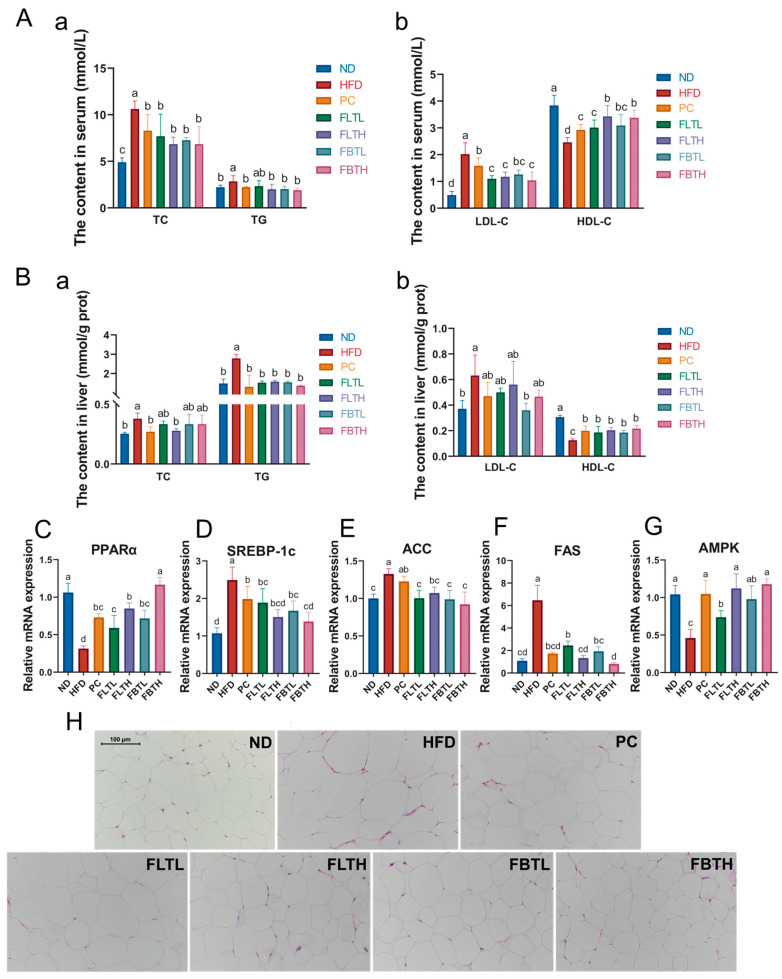
Effect of FLT and FBT on lipid metabolism in HFD-induced obese mice. (**A**) Serum lipid levels, including (**a**) TC and TG, (**b**) LDL-C and HDL-C, (**B**) liver lipid levels, including (**a**) TC and TG, (**b**) LDL-C and HDL-C. Data are presented as means ± SD (n = 10 per group). Relative expression levels of liver lipid metabolism-related genes, including (**C**) PPARα, (**D**) SREBP-1c, (**E**) ACC, (**F**) FAS, and (**G**) AMPK. (**H**) Representative H&E staining micrographs in eWAT (magnification, ×200). Data are presented as means ± SD (n = 6 per group). Significance (*p* < 0.05) among groups is expressed by different letters.

As shown in Figure 3A,B, a significant increase in TC, TG, and LDL-C levels and a remarkable decrease in HDL-C levels in the HFD group were observed (*p* < 0.05). However, after oral gavage administration with orlistat, FLT, or FBT at different doses for eight weeks, TC, TG, and LDL-C contents decreased, and HDL-C contents increased in HFD-fed mice. Histological analysis of eWAT (Figure 3H) demonstrated that HFD treatment remarkably contributed to adipocyte hypertrophy and the unevenly arranged adipocytes; however, the fat accumulation process was reversed by FLT and FBT with a dose-effected relationship. The average size of eWAT adipocytes in the HFD group was larger than that in the PC, FLT, and FBT groups, which was consistent with the results of the BW measurement (Figure 2A).

In addition, to investigate the underlying molecular mechanism regulating lipid metabolism by FLT and FBT in HFD-induced obese mice, the relative mRNA expressions of hepatic lipid metabolism-related genes, including PPARα, SREBP-1c, ACC, FAS, and AMPK, were determined by using RT-qPCR (Figure 3C–G). In the HFD group, SREBP-1c, ACC, and FAS levels significantly increased, and the relative expression levels of PPARα and AMPK decreased considerably. Compared with the HFD group, FLT or FBT administration at 400 or 800 mg/kg BW notably improved the levels of these lipid-related genes, and so did the 54 mg/kg BW orlistat supplementation. These results showed that FLT and FBT alleviated lipid metabolism disorder in HFD-induced obese mice by down-regulating the expression of lipid synthesis (SREBP-1c, ACC, and FAS)-related genes and up-regulating the expression of fatty acid oxidation (PPARα)-related genes involved in AMPK signaling pathways.

### 3.3. Effect of FLT and FBT on the Liver and Kidney Function

**Figure 4:**
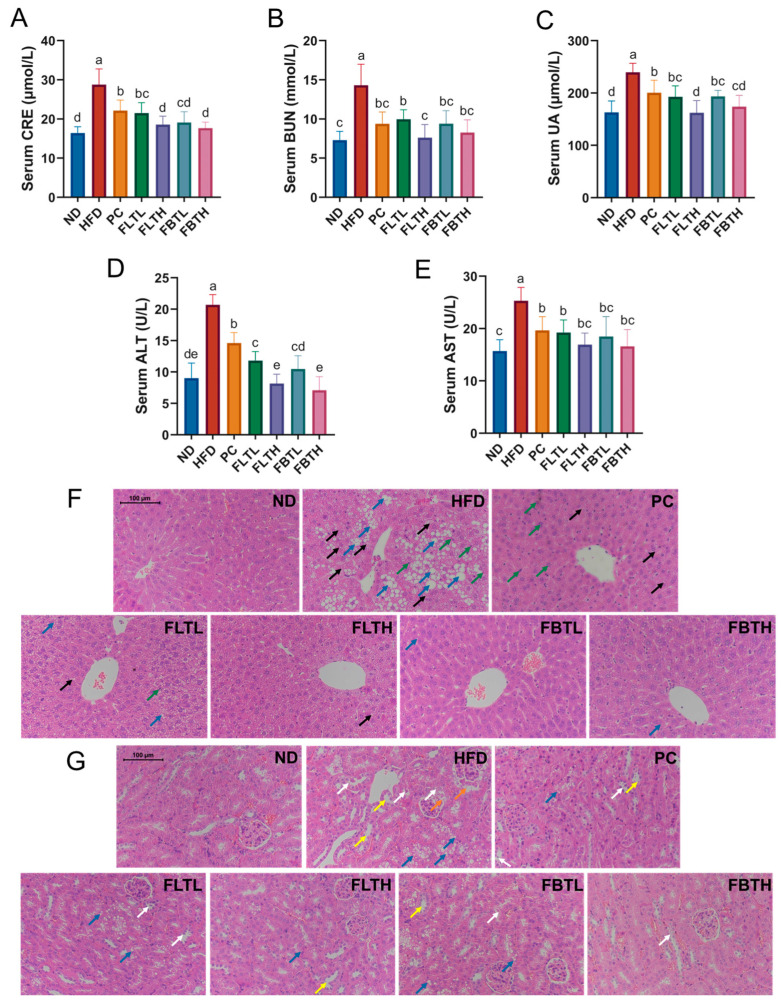
Effect of FLT and FBT on liver and kidney function and histopathologic changes in HFD-induced obese mice. (**A**) CRE, (**B**) BUN, (**C**) UA, (**D**) ALT, and (**E**) AST. Data are presented as means ± SD (n = 6 per group). Representative H&E staining micrographs in (**F**) liver and (**G**) kidney tissues (magnification, ×200). Significance (*p* < 0.05) among groups is expressed by different letters. Inflammatory cell infiltration, fatty vacuoles, renal tubular dilatation, degeneration and partial exfoliation of renal tubular epithelial cells, glomerular capsule dilatation, and ballooning degeneration are marked by green, blue, yellow, white, orange, and black arrows, respectively.

The histopathologic analysis and the determination of CRE, BUN, UA, ALT, and AST in serum were performed to assess the effect of FLT and FBT on liver and kidney function in HFD-induced obese mice (Figure 4). Histopathological analysis of the liver and kidney using H&E staining indicated that the cells of the ND group were tightly arranged and well-structured (Figure 4F,G). In contrast, inflammatory cell infiltration, liver steatosis, fatty vacuoles, and ballooning degeneration were observed in the HFD group, indicating severe fat accumulation and hepatocyte damage caused by HFD. Kidney H&E staining revealed fatty vacuoles, renal tubular dilatation, degeneration and partial exfoliation of renal tubular epithelial cells, and glomerular capsule dilatation observed in the HFD group. Interestingly, administration of orlistat, FLT and FBT alleviated these effects, especially FLT or FBT supplementation at 800 mg/kg.

AST and ALT are essential indicators in measuring the extent of liver damage, especially for ALT, which has been classified as the most sensitive indicator of liver function lesions by the World Health Organization (WHO) [32]. The ALT and AST activities were remarkably increased in the HFD group and significantly reduced after orlistat, FLT, and FBT administration (Figure 4D,E). Compared with the ND group, the serum ALT and AST activities in the HFD group dramatically increased from 9.01 and 15.72 U/L to 20.71 and 25.31 U/L, respectively. Reciprocally, supplementation of 800 mg/kg BW with FLT or FBT remarkably suppressed the ALT and AST activity increments induced by HFD, showing no statistical difference with the ND group.

The measurements of the serum kidney-associated markers (CRE, BUN, and UA) were presented in Figure 4A–C. The CRE, BUN, and UA levels were found to increase significantly in the HFD group. These findings, in conjunction with the results of kidney histopathology, suggest the presence of kidney function damage. However, this increase was reversed in the groups supplemented with orlistat, FLT, or FBT. Compared with the HFD group, in the PC, FLT, and FBT groups, CRE, BUN, and UA levels significantly decreased. Notably, the levels of CRE, BUN, and UA in the FLTH and FBTH groups remained similar to those in the ND group. These results demonstrated that FLT and FBT effectively reduced the liver and kidney dysfunction caused by HFD in mice.

### 3.4. FLT and FBT Inhibited the Oxidative Stress

**Figure 5:**
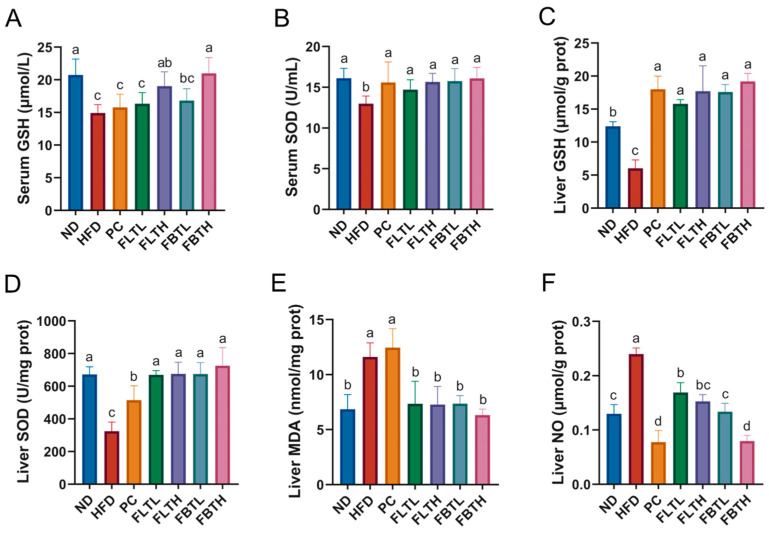
FLT and FBT ameliorated the serum and liver oxidative stress parameters in HFD-induced obese mice. (**A**) Serum GSH, (**B**) serum SOD, (**C**) liver GSH, (**D**) liver SOD, (**E**) liver MDA, and (**F**) liver NO. Data are presented as means ± SD (n = 6 per group). Significance (*p* < 0.05) among groups is expressed by different letters.

As shown in Figure 5, this study measured the activities of endogenous antioxidant enzymes (GSH and SOD) and the levels of lipid peroxidation biomarkers (MDA and NO) to evaluate the mitigating impact of FLT and FBT against oxidative damage in HFD-induced obese mice. Compared with the ND group, the GSH and SOD activities in the serum and liver in the HFD group were all significantly decreased (*p* < 0.05), and the MDA and NO levels in the liver were significantly increased (*p* < 0.05). In comparison, FLT and FBT supplementation, especially at high doses, significantly enhanced GSH and SOD activities and decreased MDA and NO levels. Consuming FLT or FBT at 800 mg/kg BW dramatically inhibited the oxidative damage induced by HFD and restored antioxidant capacity to the levels in the ND group. Overall, FLTH or FBTH treatment was more effective than orlistat treatment in countering HFD-induced oxidative damage. Besides, no statistical difference in MDA levels was measured between the HFD and PC groups. These results revealed that FLT and FBT could repair the antioxidant defense system in HFD-fed mice.

### 3.5. FLT and FBT Improved the Hepatic Inflammatory Response

**Figure 6:**
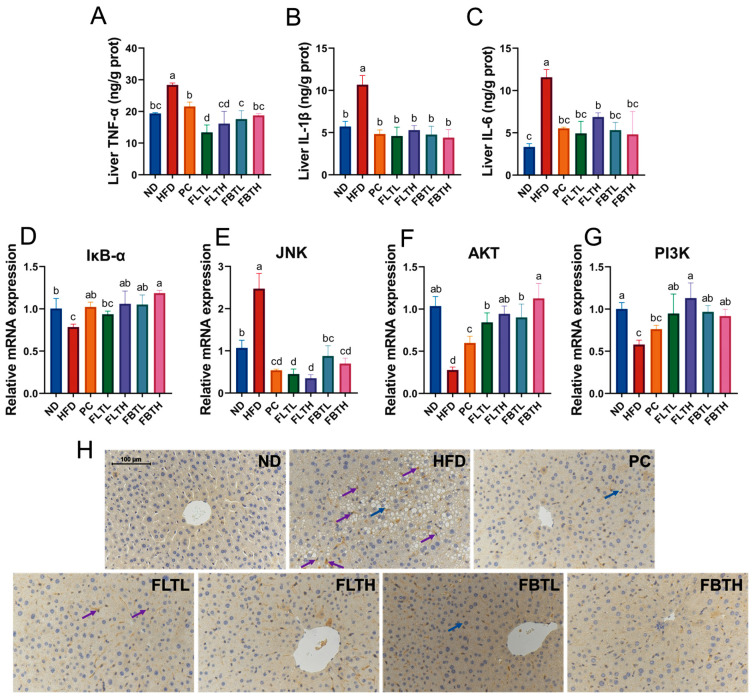
FLT and FBT ameliorated the liver inflammation in high-fat diet-induced obese mice. The levels of liver (**A**) TNF-α, (**B**) IL-1β, and (**C**) IL-6. Relative mRNA expression levels of inflammatory factors in liver, including (**D**) IκB-α, (**E**) JNK, (**F**) AKT, and (**G**) PI3K. (**H**) Immunohistochemistry of the liver for NF-κB p65. Data are presented as means ± SD (n = 3 per group). Significance (*p* < 0.05) among groups is expressed by different letters. Fatty vacuoles and positive cells are marked by blue and purple arrows.

The potential possibility of FLT or FBT supplementation in alleviating the inflammatory reaction in HFD-induced mice was investigated by measuring several inflammatory cytokines and mRNA expression levels of several inflammation-related genes. As shown in Figure 6A–C, the hepatic TNF-α, IL-1β, and IL-6 levels in the HFD group exhibited a notable increase (*p* < 0.05), suggesting that inflammation has developed to some extent in the obese mice. Nevertheless, administration with FLT, FBT, or orlistat for eight weeks significantly suppressed the liver’s TNF-α, IL-1β, and IL-6 expression levels (*p* < 0.05), thus alleviating the inflammation induced by obesity. Figure 6H illustrates the hepatic expression levels of NF-κB p65 measured by immunohistochemical assay, which revealed that the NF-κB p65 level was increased in the HFD group. Still, the expression level of NF-κB p65 was dramatically down-regulated by supplementation with FLT, FBT, and orlistat.

In addition, the mRNA expression levels of inflammation-related genes were measured using RT-qPCR. As displayed in Figure 6D–G, the improvement of FLT and FBT on levels of IκB-α, JNK, AKT, and PI3K in the liver tissues was investigated. In the mice of the HFD group, the levels of IκB-α, AKT, and PI3K were notably down-regulated (*p* < 0.05), whereas the JNK level was up-regulated. However, after FLT, FBT, or orlistat supplementation for eight weeks, the levels of these genes in the PC, FLTL, FLTH, FBTL, and FBTH groups were close to those in the ND group. These results demonstrated that the inflammatory response in HFD-fed mice could be attenuated by supplementation with FLT or FBT.

### 3.6. FLT and FBT Altered the Structure and Composition of the Gut Microbiota

**Figure 7:**
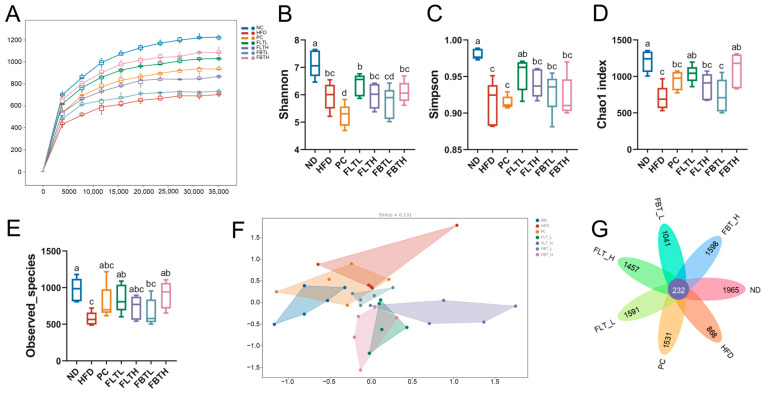
Effect of FLT and FBT on the gut microbiota structure in the colon contents collected from HFD-induced obese mice. (**A**) Rarefaction curves, (**B**) Shannon index, (**C**) Simpson index, (**D**) Chao1 index, (**E**) observed species, (**F**) NMDS, and (**G**) Venn diagram of OTUs. Significance (*p* < 0.05) among groups is expressed by different letters.

To clarify the gut microbiota structure variation after FLT and FBT administration in HFD-induced mice, colonic contents collected from seven groups (n = 5 per group) were subjected to 16S rRNA high-throughput sequencing. Alpha diversity was performed on the obtained OUTs abundance matrix (Figure 7). The rarefaction curves of all groups gradually flattened out, indicating that the present species richness was available. In the HFD group, the Shannon index, Simpson index, Chao1 index, and observed species decreased. However, the Simpson index, Chao1 index, and observed species were dramatically increased (*p* < 0.05) after 400 mg/kg BW FLT (FLTL group) administration for eight weeks. In addition, 800 mg/kg BW FBT (FBTH group) administration remarkably increased the Chao1 index and observed species (*p* < 0.05). This result demonstrated that FLT and FBT could enrich gut microbiota diversity. Beta diversity, represented by NMDS, indicated a distinct difference in the gut microbiota structure that was demonstrated across the different treatment groups, where the HFD group was significantly separated from the ND, FLTL, FLTH, FBTL, and FBTH groups. The Venn diagram shows that 232 common OUTs were found in all groups, and the ND group possesses the highest species diversity among all groups, followed by the FBTH group.

**Figure 8:**
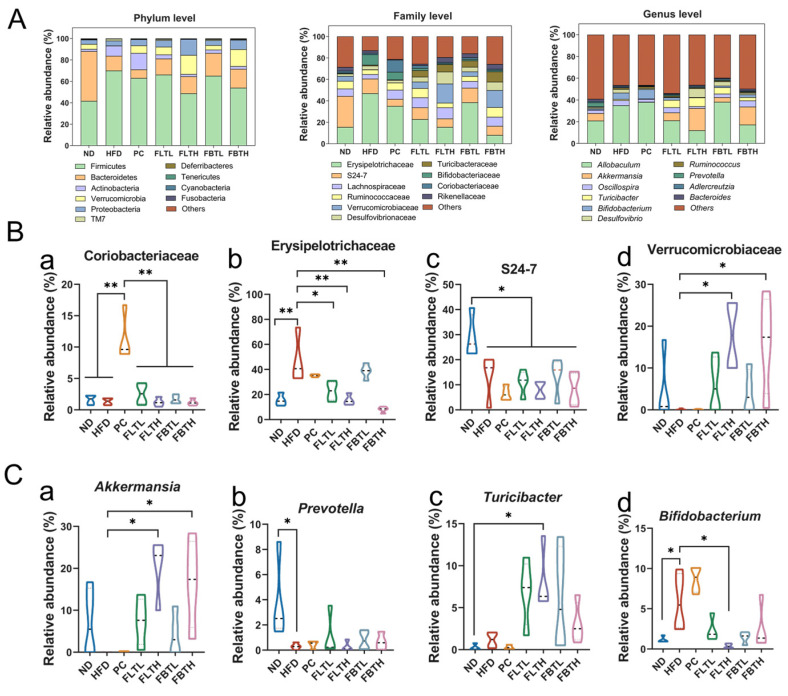

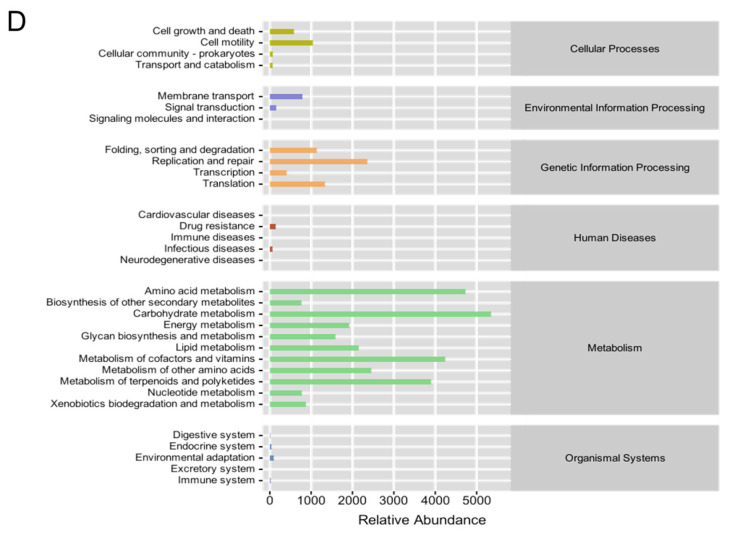
Effect of FLT and FBT on the gut microbiota composition in the colon contents collected from HFD-induced obese mice. (**A**) Relative abundance of gut microbiota at the phylum level, family level, and genus level. (**B**) Relative abundance of the key bacteria at the family level, including (**a**) Coriobacteriaceae, (**b**) Erysipelotrichaceae, (**c**) S24-7, and (**d**) Verrucomicrobiaceae. (**C**) Relative abundance of the key bacteria at the genus level, including (**a**) *Akkermansia*, (**b**) *Prevotella*, (**c**) *Turicibacter*, and (**d**) *Bifidobacterium*. (**D**) KEGG pathway enrichment analysis. * *p* < 0.05, ** *p* < 0.001.

Moreover, as shown in Figure 8A–C, the differences in the relative abundance of the predominant microbiota were analyzed to investigate the specific changes in gut microbiota compositions across different treatment groups. At the phylum level (Figure 8A), Firmicutes, Bacteroidetes, Actinobacteria, Verrucomicrobia, and Proteobacteria were the dominant taxa in all groups, especially Firmicutes and Bacteroidetes. The levels of Firmicutes and Actinobacteria in the HFD group were much higher than those in the ND group. A remarkable decline of Bacteroidetes and Verrucomicrobia, especially Verrucomicrobia, was induced by HFD. Interestingly, FLT and FBT supplementation could reverse such changes and was more effective than orlistat supplementation to some extent. At the family level (Figure 8B), compared with the HFD and PC groups, FLT and FBT administration reduced the Erysipelotrichaceae abundance and increased Verrucomicrobiaceae abundance with a dose-effected relationship. The comparative analysis reflected that HFD significantly lowered the level of S24-7, and the orlistat administration induced a notable increase in Coriobacteriaceae. At the genus level, the total account of *Allobaculum* and *Akkermansia* was the highest of all groups (Figure 8A). Figure 8C details the microbiota that is significantly different among treatment groups at the genus level. Compared with the other groups, the *Akkermansia* level in the HFD and PC groups was considerably lower, indicating FLT and FBT can increase the abundance of *Akkermansia* even to higher levels than that in the ND group by 800 mg/kg administration. Conversely, among all groups, the *Bifidobacterium* abundance in the HFD and PC groups significantly increased. The *Turicibacter* abundance showed no significant difference among the ND, HFD, and PC groups, but it was dramatically increased by FLT and FBT supplementation. However, FLT, FBT, or orlistat supplementation did not alleviate the decreased abundance of *Prevotella* induced by HFD. These findings demonstrated that FLT and FBT administration contributed to a remarkable effect on gut microbiota composition.

### 3.7. The Potential Regulation Prediction of FLT and FBT on Metabolic Pathways

To explore the underlying mechanisms of FLT and FBT modulating the gut microbiota and thus ameliorating the obesity-related symptoms induced by HFD, the KEGG enrichment analysis was carried out using PICRUSt2 (Figure 8D). The KEGG enrichment analysis showed that “metabolism” was the most enriched category in this study. Notably, among all enriched pathways, more gut microbiota was annotated into the carbohydrate, amino acid, lipid, and energy metabolism, indicating that supplementation with FLT or FBT can alleviate the metabolic disorders owing to HFD by improving the gut microbiota balance.

## 4. Discussion

Owing to the change in lifestyle under the current economic conditions, obesity and its metabolic complications as a consequence of the high-fat diet have become a severe public health issue worldwide. Fu tea, as a dietary supplement containing polyphenols and polysaccharides, is found to have health-promoting and chronic metabolic disease prevention potential [33]. Therefore, the underlying mechanism of the effects of FLT and FBT administration on HFD-induced obese mice was explored in this study.

### 4.1. FLT and FBT Alleviated Lipid Homeostasis and Oxidative Stress

The present studies demonstrated that HFD-induced obesity is generally accompanied by weight gain, adipocyte hypertrophy, glucose intolerance, and dyslipidemia. Similar to previous studies, the current findings again indicated that prolonged exposure to HFD resulted in obesity and hypertriglyceridemia. HDL, an important factor in promoting cholesterol metabolism [34], was notably elevated by FLT or FBT supplementation in HFD-fed mice. Du et al. [33] reported that daily FBT administration (400 mg/kg) inhibited the hepatic steatosis and adipocyte size increment associated with HFD [23], which aligns with the findings of our current investigation. The levels of hepatic lipid metabolism-related genes were measured (Figure 3), as the liver is a vital organ involved in lipid homeostasis [35]. In the current study, FLT and FBT administration (400, 800 mg/kg BW) improved lipid metabolism in HFD-induced obese mice by downregulating the levels of fatty acid synthesis-related genes (SREBP-1c, ACC, and FAS) and upregulating the levels of fatty acid oxidation-related genes (PPARα and AMPK). These results demonstrate that FLT and FBT inhibited fatty acid formation and promoted fatty acid oxidation by regulating the lipid metabolism-related genes involved in the AMPK pathway, thereby alleviating lipid metabolism disorders induced by HFD.

The oxidative stress-related factors were also assessed, as they play essential roles in developing metabolism disorders caused by obesity [36]. Obese mice fed HFD for a long period of time may more likely develop lipid peroxidation and oxidative stress. SOD, GSH, MDA, and NO are essential indicators of oxidative stress [32]. In the present work, FLT or FBT supplementation remarkably increased the serum and liver GSH and SOD activities while lowering the levels of liver MDA and NO (Figure 5), which may be attributed to reduced free radical production and enhanced antioxidant capacity, suggesting that FLT or FBT administration could prevent HFD-induced oxidative stress [37].

### 4.2. FLT and FBT Inhibited the Organ Damage and Inflammatory Response

Current research suggests that obesity induced by a long-term HFD may damage kidney and liver tissue [36,38]. CRE, BUN, and UA have been widely used as screening tools for preliminary assessment of kidney health status [39]. Hung et al. [38] observed increased levels of CRE, BUN, and UA in HFD-induced obese mice, indicating potential renal damage, in agreement with the results of our study. Elevated serum CRE, BUN, and UA levels combined with histopathologic changes in kidney tissue suggest different degrees of renal structural and functional injuries in HFD-fed mice. However, these symptoms were reversed (Figure 4) after the intervention of FLT or FBT, indicating FLT or FBT supplementation could prevent the deterioration of renal function caused by HFD.

Serum AST and ALT levels have been proven valuable in assessing liver function [36]. Herein, FLT or FBT administration dramatically reduced HFD-induced elevation of serum AST and ALT levels (Figure 4D,E) and improved hepatic histopathology changes induced by HFD (Figure 4F), indicating that FLT and FBT were able to attenuate liver injury in HFD-fed mice. Additionally, hepatic injury is commonly accompanied by inflammation [40]. Chronic inflammation is an essential factor leading to PI3K/AKT insulin signaling pathway damage [41], which is a primary trigger of insulin resistance (IR) [42]. The JNK/NF-κB [42] and IκB-α/NF-κB [43] pathways have been demonstrated to mediate the levels of various inflammatory factors, and activation of them promotes the secretion of a variety of pro-inflammatory cytokines [44]. In this study, a notable reduction in mRNA expression levels of hepatic PI3K and AKT was observed in the HFD group (Figure 6F,G). Moreover, in the HFD group, the levels of hepatic inflammatory-related genes (JNK and IκB-α) and inflammatory cytokines (TNF-α, IL-1β, and IL-6) were increased. However, after FLT or FBT administration for eight weeks, the PI3K/AKT pathway was activated, thus inhibiting the inflammatory response. In addition, FLT and FBT effectively inhibited the expression of hepatic NF-κB, as measured by immunohistochemical analysis (Figure 6H). Notably, the alleviative effects of FLT and FBT on hepatic injury and inflammatory response were consistent with histopathological examination. Overall, FLT and FBT could attenuate HFD-induced liver injury by modulating liver function, alleviating oxidative stress, and suppressing inflammation.

### 4.3. FLT and FBT Exerted Anti-Obesity Effects by Maintaining the Balance of Gut Microbiota

The gut microbiota, considered a “virtual organ”, is essential in the regulation of metabolic homeostasis and energy balance. Reportedly, a prolonged HFD could disrupt the gut microbiota structure and composition [45]. Further analysis of the present study revealed that FLT and FBT improved the negative impact of HFD on the gut microbiota. At the family level, FLT and FBT significantly reversed the increased Erysipelotrichaceae abundance and decreased Verrucomicrobiaceae abundance in the HFD-induced mice. Interestingly, the relative abundance of Coriobacteriaceae in the PC group was higher than that in other groups; none of the treatments showed positive effects on the decreased abundance of S24-7 induced by HFD. Chen et al. [46] observed that the elevated presence of Erysipelotrichaceae in the mice fed by HFD related to lipidemic profiles [47] could be reduced by the administration of FBT polysaccharides, especially by the high dosage of FBT administration, consistent with the present data. The abundance of Verrucomicrobiaceae, significantly positively related to immune traits reported by Ding et al. [48], was increased by FLT and FBT administration with a dose-effected relationship compared with the HFD group in this work. In contrast, orlistat intervention did not increase the Verrucomicrobiaceae abundance in the mice fed by HFD, suggesting that orlistat intervention did not alleviate the immunosuppression induced by HFD. Coriobacteriaceae, belonging to the Actinobacteria phylum, was found to have pathogenic potential and positively correlated with the development of inflammatory bowel disease [49], indicating that orlistat intervention in this study might induce the development of enterocolitis, whereas FLT and FBT did not.

At the genus level, FLT and FBT notably increased the abundance of *Akkermansia* and *Turicibacter* while reducing the abundance of *Bifidobacterium* to levels comparable to those in the ND group. In addition, no significant effect on *Prevotella* abundance after FLT and FBT treatments was observed compared with the HFD group, and the orlistat supplementation did not effectively reduce the abundance of *Bifidobacterium*. As Wu et al. reported, *Akkermansia*, belonging to Verrucomicrobiaceae, could improve glucose homeostasis and insulin sensitivity, thereby reducing IR and intestinal inflammation and improving host health [2]. The present data showed that FLT and FBT dramatically increased the relative abundance of *Akkermansia* compared with orlistat intervention. It is reported that *Turicibacter* is an anti-inflammatory taxon that exerts a pivotal function in the inflammatory signaling pathway [50]. The present study indicated that FLT and FBT treatment could increase *Turicibacter* abundance. *Prevotella*, observed with carbohydrate consumption [51], was reported to be associated with alleviating obesity and metabolic disorders [52]. Du et al. [19] demonstrated that FBT could enhance the abundance of *Prevotella*, the same as the present data. Du et al. [53] proved that the increased abundance of *Bifidobacterium* in the mice fed by HFD, likely to be associated with polysaccharide and other complex carbohydrates consumption and indicating the gut microbiota imbalance or inflammatory response, was reduced by blueberry anthocyanins and blackberry anthocyanins administration, which is similar to this study.

KEGG analysis of the detected gut microbiota revealed that carbohydrate, amino acid, and lipid metabolism were dominantly enriched metabolic pathways after different treatments. Long-term HFD could suppress gluconeogenesis, promote glycolysis [54], and thus affect carbohydrate metabolism, whereas FLT or FBT administration reversed this state to some extent. Imbalances in amino acid metabolism caused by HFD can result in various health problems. Some amino acids may be participating in regulating energy homeostasis, nutrient metabolism, and immunity via the PI3K/AKT pathway, which is associated with the development of IR [55]. Moreover, obesity has been demonstrated to induce lipid metabolism disorders, a critical and complicated biochemical reaction [56]. Therefore, FLT and FBT administration may alleviate metabolic disorders resulting from HFD by modulating gut microbial homeostasis. Shortly, additional research exploring the impacts of FLT and FBT on the obtained microbiota and metabolites in specific metabolic pathways using genomics and metabolomics methods is imperative to gain more precise and comprehensive insights.

## 5. Conclusions

This work demonstrated that the beneficial effects of FLT on alleviating HFD-induced obesity were almost the same as those of FBT. The main mechanisms of FLT or FBT administration at 400 and 800 mg/kg BW in reducing fat accumulation, improving lipid profile, inhibiting oxidative stress, ameliorating inflammatory response, and decreasing liver and kidney damage may be attributed to the regulation of lipid metabolism-related genes and inflammation-related genes as well as the reshaped gut microbiota. Moreover, diet pill (orlistat) administration showed no significant reverse effect on alleviating gut microbiota dysbiosis. Therefore, FLT and FBT are excellent dietary supplements to prevent obesity and its complications. In particular, FLT may be more consumed because of its short processing time and convenient consumption. Still, further investigation is warranted to fully elucidate the underlying molecular mechanisms and explore the potential of FLT and FBT as therapeutic interventions for obesity and related metabolic diseases.

## Data Availability

The original contributions presented in the study are included in the article/supplementary material, further inquiries can be directed to the corresponding author.

## Supplementary Materials

The following supporting information can be downloaded at: https://www.mdpi.com/article/10.3390/foods13020206/s1, Table S1: Primer sequences and the PCR product amplified fragments used in RT-qPCR.
