# Supplementary material for: Fu Loose Tea Administration Ameliorates Obesity in High-Fat Diet-Fed C57BL/6J Mice: A Comparison with Fu Brick Tea and Orlistat
Source: Foods. 2024 Jan 9;13(2):206. doi: 10.3390/foods13020206 (PMC10815023; doi:10.3390/foods13020206)
Supplement: Supplementary file 1 [file foods-13-00206-s001.zip › foods-2782991-supplementary.pdf]

Table S1. Primer sequences and the PCR product amplified fragments used in RT-qPCR

Genes	Accession no.	Sequences (**5′→3′**)
SREBP-1c	NM_001313979.1	CCATCGACTACATCCGCTTCTT
		CAGGTCCTTCAGTGATTTGCTTT
ACC	NM_133360.2	TTTGTTTGGTCGTGACTGCTCTG
		AGGATGTTCAACCTGTAGCCGAG
FAS	NM_001146708.1	TGTCCTGCCTCTGGTGCTTG
		GCAAAATGGGCCTCCTTGATAT
PPARα	NM_011144.6	CACTACGGAGTTCACGCATGT
		GTGACATCCCGACAGACAGGC
AMPK	NM_001013367.3	AACCTGAGAACGTCCTGCTTGAT
		CTTCCTGAAATGACTTCTGGTGC
JNK	NM_001310452.1	GCCATTTCAGAATCAGACCCAT
		CCCGATGAATAATTCCAGCAGA
PI3K	NM_001024955.2	AAACTCCGAGACACTGCTGATG
		GCTGGTATTTGGACACTGGGTA
IκB-α	NM_010907.2	CACTTGGTGACTTTGGGTGCT
		GCTGTATCCGGGTACTTGGG
AKT	NM_009652.3	CTTCCTCCTCAAGAACGATGGC
		TGTCTTCATCAGCTGGCATTGT
GAPDH	NM_008084.2	CCTCGTCCCGTAGACAAAATG
		TGAGGTCAATGAAGGGGTCGT

SREBP-1c, sterol regulatory element binding protein-1c; ACC, acetyl-CoA carboxylase, FAS, fatty acid synthase; PPARα, peroxisome proliferator-activated receptor-alpha; AMPK, AMP-activated protein kinase; JNK, c-Jun-N-terminal kinase; PI3K, phosphatidylinositol-3-hydroxy kinase; IκB-α, IkappaB-alpha; AKT, protein kinase B; GAPDH, Glyceraldehyde-3-phosphate dehydrogenase.
